# Rosette Trajectory MRI Reconstruction with Vision Transformers

**DOI:** 10.3390/tomography11040041

**Published:** 2025-04-01

**Authors:** Muhammed Fikret Yalcinbas, Cengizhan Ozturk, Onur Ozyurt, Uzay E. Emir, Ulas Bagci

**Affiliations:** 1 Institute of Biomedical Engineering, Boğaziçi University, Istanbul 34684, Turkey; cozturk@bogazici.edu.tr; 2 Center for Targeted Therapy Technologies (CT3), Boğaziçi University, Istanbul 34984, Turkey; 3 Wolfson Brain Imaging Centre, Department of Clinical Neurosciences, University of Cambridge, Cambridge CB2-0QQ, UK; oo309@medschl.cam.ac.uk; 4 Department of Radiology, University of North Carolina at Chapel Hill, Chapel Hill, NC 27514, USA; uzay_emir@med.unc.edu; 5 Machine and Hybrid Intelligence Lab, Northwestern University, Chicago, IL 60611, USA; ulas.bagci@northwestern.edu

**Keywords:** MRI, machine learning, medical imaging

## Abstract

Introduction: An efficient pipeline for rosette trajectory magnetic resonance imaging reconstruction is proposed, combining the inverse Fourier transform with a vision transformer (ViT) network enhanced with a convolutional layer. This method addresses the challenges of reconstructing high-quality images from non-Cartesian data by leveraging the ViT’s ability to handle complex spatial dependencies without extensive preprocessing. Materials and Methods: The inverse fast Fourier transform provides a robust initial approximation, which is refined by the ViT network to produce high-fidelity images. Results and Discussion: This approach outperforms established deep learning techniques for normalized root mean squared error, peak signal-to-noise ratio, and entropy-based image quality scores; offers better runtime performance; and remains competitive with respect to other metrics.

## 1. Introduction

### 1.1. MRI, Cartesian and Non-Cartesian

Magnetic resonance imaging (MRI) is a crucial diagnostic tool in modern medicine, capable of providing detailed images of anatomical structures [1]. MRI data are collected in k-space, where signals are recorded based on their spatial frequency and phase information [2]. These k-space data are then transformed into the spatial domain through the Fourier transform [3]. The resulting images provide high-resolution, contrast-rich views of anatomical structures, aiding in accurate diagnosis and treatment planning. The Cartesian trajectory of k-space corresponds to a sampling scheme in which data are collected along regular grid lines. While such fully sampled images are ideal, some degree of undersampling is required to reduce scan times and minimize patient discomfort [4]. Non-Cartesian trajectories, such as radial or spiral paths, yield improved image quality compared to Cartesian undersampling, allowing faster MRI acquisition via efficient k-space coverage [5].

### 1.2. Cartesian K-Space MRI Reconstruction Methods

Traditional Cartesian reconstruction methods involve the direct application of the inverse fast Fourier transform (IFFT) to uniformly sampled k-space data. Despite its widespread use, standard FFT-based reconstruction is prone to artifacts from motion and undersampling. Compressed sensing has emerged as a key alternative, exploiting the sparsity of undersampled data to reconstruct images by solving an optimization problem that enforces sparsity constraints, leading to high-quality reconstructions with reduced scan times [6]. Compressed sensing is often used in conjunction with parallel imaging, the combination being abbreviated as PICS. Parallel imaging techniques, such as sensitivity encoding (SENSE) and generalized autocalibrating partially parallel acquisitions (GRAPPA), leverage the spatial sensitivity profiles of multiple receiver coils to accelerate MRI acquisitions [7].

Deep learning methods have had success in reconstructing undersampled Cartesian MRI [8,9,10,11], improving upon classical techniques at high accelerations by allowing greater complexity in the employed model [12] and extracting complex mappings from training data [13]. Architectures such as convolutional neural networks (CNNs) and generative adversarial networks have been employed to restore missing or corrupted k-space data, leveraging large datasets to enhance reconstruction speed and accuracy [14,15].

### 1.3. Non-Cartesian K-Space MRI Reconstruction Methods

Reconstructing high-quality images from non-Cartesian data is challenging, as traditional Fourier-based methods struggle to handle the irregularity and gaps in the sampling pattern [16]. Standard non-Cartesian reconstruction methods typically involve arranging the data onto a Cartesian grid before applying the FFT, a process known as gridding [17]. The sparsity of non-Cartesian data also makes it a suitable candidate for compressed sensing, non-Cartesian GRAPPA, and SENSE [5]. Methods such as compressed sensing overcome the limitations of Fourier techniques by balancing fidelity to acquired data with the assumption of sparsity in the image domain [7].

Deep neural networks gained traction in this problem [18] due to their ability to handle non-Cartesian data without the need for regridding. Hammernik et al. [14] employed VarNet for the reconstruction of complex multi-channel MR data, leveraging its ability to seamlessly combine variational inference techniques with deep learning to achieve impressive performance in reconstruction tasks [19]. Aggarwal et al. introduced MoDL [20], an integration of model-based and deep learning approaches. The MoDL architecture applies iterative unrolling optimization algorithms within a deep learning framework that enables accurate and efficient reconstructions. Vision transformers have also demonstrated state-of-the-art performance in non-Cartesian MRI reconstruction [21]. Unlike traditional CNNs, which rely on convolutional kernels to process spatial information, ViTs decompose the input data into a sequence of patches and apply transformer models to learn complex representations [22]. Table 1 summarizes the advantages and disadvantages of the reconstruction techniques discussed. It should be noted that non-Cartesian reconstruction with deep neural networks can require specialized algorithms and additional preprocessing [23]. These limitations, combined with the need for domain-specific expertise to implement and optimize non-Cartesian deep learning techniques, present challenges to their practical application. This is particularly relevant for accelerated acquisition scenarios, which are essential for further reducing scan times while maintaining high image quality.

### 1.4. The Rosette Trajectory

The rosette trajectory, a non-Cartesian trajectory that traces a petal-like path through k-space, provides particularly effective k-space coverage [25]. The efficient sampling pattern of the rosette trajectory can yield a higher signal-to-noise ratio (SNR) [26] and shorter scan times [27] compared to spiral or radial trajectories for MRI acquisition under high acceleration factors. Standard reconstruction techniques for rosette k-space involve adapting traditional gridding methods to handle the unique structure of the trajectory, including the development of dedicated density compensation functions (DCF) to account for the varying sampling density [28].

Rosette has been shown to work well alongside CS and high acceleration factors [25]. Mahmud et al. achieve good performance with an acceleration factor of 6 in 7 Tesla CS rosette spinal cord imaging [29]. Li et al. use rosette and CS to achieve 10 percent better results than radial and spiral trajectories at an acceleration factor of 10 [26]. Alcicek et al. and Bozymski et al. demonstrate the feasibility of using a novel rosette trajectory with CS to achieve patient-friendly and high-resolution magnetic resonance spectroscopy imaging [30,31].

While the rosette trajectory is a promising candidate for high-acceleration reconstruction, training on raw MRI data is computationally expensive. In order to achieve flexible high-accuracy reconstruction for accelerated rosette imaging with good runtime performance, we utilize the direct Fourier transform to approximate the final result and the ViT network to transform this approximation into the expected image. The addition of a convolutional layer improves the reconstructed image by removing artifacts that arise from the ViT network. This approach enhances the reconstruction of rosette trajectory MRI, making it not only faster but also more adaptable across different imaging scenarios. As a benchmark, we apply VarNet [14] and MoDL [20].

## 2. Materials and Methods

### 2.1. Method Overview

**Figure 1:**
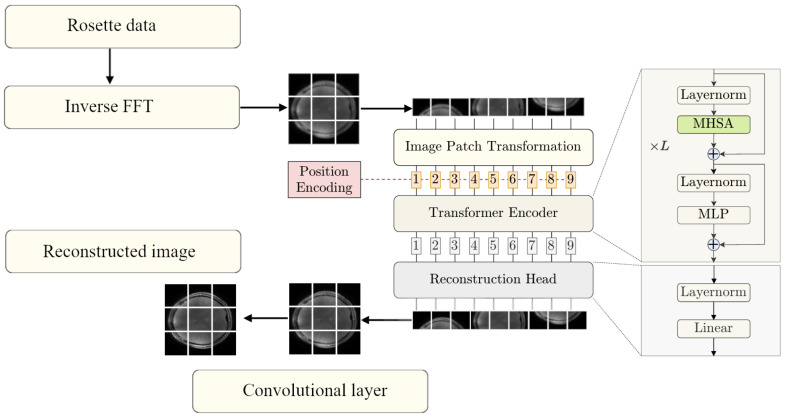
Pipeline of IFFT to ViT reconstruction (ViT stage adapted from Lin and Heckel [21]).

The method proposed in this work utilizes a strategy that combines traditional and modern deep learning techniques. The pipeline, shown in Figure 1, operates in two key phases: an initial approximation phase and a refinement phase. During the initial approximation phase, the IFFT is applied to multi-coil rosette-sampled k-space data, after which the resulting coil images are combined through root square sum to produce a rough image. This step, though computationally simple, introduces imprecision and noise due to the undersampled and non-uniform nature of the k-space trajectory. In the refinement phase, the rough image is passed to the ViT network, which has been trained with our data to predict the PICS reconstruction.

### 2.2. Vision Transformer

The network architecture of the employed ViT is based on the accelerated MRI reconstruction work by Lin and Heckel [21], adapted for approximating the compressed sensing algorithm for MRI reconstruction. Their model is based on the ViT, originally proposed by Dosovitskiy et al. [32] for image classification tasks. This involves processing the input image as a sequence of patches in order to apply the transformer encoder, which was designed for sequential data. A trainable linear transformation maps each patch to a d-dimensional feature vector known as a patch embedding. To compensate for the lack of positional information, learnable position embeddings are used to encode the absolute position. Finally, a classification token is added to the beginning of the sequence. The encoder consists of N encoder layers, each containing a multi-head self-attention (MHSA) block and a multilayer perceptron block that transforms each feature vector independently. The Swin ViT block, used in the Swin architecture, replaces the MHSA block with a window-based self-attention (WBSA) block that parses the region of attention in sections [24]. In both cases, layer normalization is applied before each block, and residual connections are added after each block for stable training. The output representation of the classification token is used for the final classification of the input image. The ViT architecture by Lin and Heckel adapts this system for image reconstruction by removing the classification token and replacing the classification head with a reconstruction head tailored for mapping the transformer output back to a visual image [21]. Unlike previous approaches that combine transformers with convolutions [33], this architecture uses only the standard transformer encoder.

**Figure 2:**
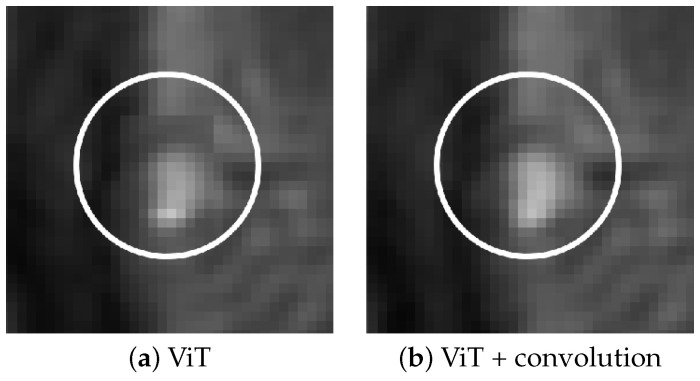
Effect of convolution in removing patch artifacts. Subfigure (**a**) shows ViT reconstruction without convolution, (**b**) shows reconstruction with the convolutional layer added.

In our experiments, the MHSA ViT model produced artifacts in the form of miscolored patches with defined boundaries at regions of high contrast, irrespective of training augmentation. The inclusion of the convolutional layer corrects this issue. Figure 2 shows the improvement granted by the addition of the layer.

### 2.3. Dataset and Preprocessing

The experimental dataset comprises 6 sets of 500 T1-weighted time series axial brain MRI scans acquired using the rosette k-space trajectory. Imaging was performed on a Siemens MAGNETOM Terra 7 T system with a Nova Medical 8Tx/32Rx head coil. The acquisition details [34] are as follows:

- Repetition time (TR): 2.4 s;
- Echo time (TE) (dual): 1 and 9 milliseconds;
- Acceleration factor: 4;
- Total petals: 189;
- $K_{max}$: 1000/m;
- $w_{1}$: 400 Hz;
- $w_{2}$: 400 Hz;
- Nominal in-plane resolution: 0.468 mm;
- Slice thickness: 2 mm;
- Flip angle: 7 degrees;
- Image resolution: 512 × 512.

We employed both augmented and non-augmented datasets for ViT training and evaluation. Reference images for comparison were generated using the PICS algorithm implemented through the Berkeley Advanced Reconstruction Toolbox (BART) [35] (https://mrirecon.github.io/bart) (accessed on 20 June 2024). The k-space data were converted to an initial approximation of the final image using the IFFT. These intermediate images are then used to train the network to minimize the difference between such inputs and the CS reference.

Four training sets were used for training, and one set was used for validation, with 500 scans in each set. For singly augmented ViT, this results in 4000 inputs, and for triple augmentation, this number becomes 8000.

The augmentations used for ViT training data include:

- Random horizontal flip, probability = 0.5;
- Random vertical flip, probability = 0.5;
- Random rotation, 0 to 180 degrees;
- Color jitter, brightness/contrast/saturation, range = 0.8 to 1.2;
- Random resized crop, scale = 0.3 to 1.1.

By simulating various imaging conditions during training, the network learns to extract meaningful features while remaining flexible to variations in patient anatomies, imaging conditions, and acquisition scenarios.

### 2.4. Evaluation Methods

Reconstruction performance was assessed using several image quality metrics. Table 2 shows the respective formulae. The metrics are applied to six randomly generated 50 × 50 pixel patches (biased towards the center to avoid empty space) and averaged to generate the final metric score.

- The structural similarity index measure (SSIM) measures image similarity between a reference image and a processed image [36]. Higher scores are preferred.
- Normalized root mean square error (NRMSE) in the context of image quality is the square root of the mean squared error [37] between two images normalized by the sum of the observed values. Lower error is preferred.
- Normalized mutual information (NMI) measures shared information, where the scale between no mutual information and full correlation is given as 0 to 1 [38].
- Relative contrast is the ratio between the difference in maximum and minimum intensity and the sum of the same values.
- Peak signal-to-noise ratio (PSNR) measures the ratio between the maximum possible pixel value and the noise power [39]. Higher PSNR values indicate better image quality.
- Shannon entropy quantifies the information content of an image using a measure of uncertainty [40].
- The entropy focus criterion (EFC) provides an estimate of corruption and blurring in terms of energy—lower values are preferred [41].

SSIM, NRMSE, and NMI are used to investigate the fidelity of the reconstructed image in terms of structure, intensity, and shared information. Relative contrast and PSNR were chosen to measure the individual quality of the resulting image by quantifying signal strength and intensity range. Shannon entropy was used to assess the addition or loss of information, neither of which is desirable. The EFC criterion is useful as an indicator of clarity for an individual image.

### 2.5. Visualization

The reconstructed images were bias field corrected with the open source Advanced Normalization Tools toolbox (https://github.com/ANTsX (accessed on 12 November 2024)).

### 2.6. Training Procedure

The ViT training was completed using the PyTorch (2.5.1) library (created at Facebook, now Meta AI) on an Nvidia A6000 GPU. For gradient descent, the Adam optimizer [42] was used with a maximum learning rate of 0.0003. The “1cycle” learning rate scheduler [43] was used alongside Adam to adjust the learning rate. A patch size of 10 × 10 pixels was used, defining the size of the square segments into which the input image is divided. The depth of the sequential layers of self-attention and feedforward networks was set as 10, while the number of attention heads were set at 16. The embedding dimension was set at 80, partly due to memory concerns. The convolutional layer is set at a kernel size of 3 and stride of 1 to preserve the output image dimension. The small kernel size preserves the quality of the image while correcting boundary artifacts generated by the ViT. The networks converged in 15 to 20 epochs, at which point the training was stopped. Ground truth is defined as the PICS reconstruction. The benchmark VarNet and MoDL networks are used with a setup informed by Blumenthal et al. [35], with batch size set at 20.

## 3. Results

### 3.1. Image Scores

Table 3 shows various image quality metric results for the ViT pipeline against VarNet and ModL. The highest SSIM score is achieved by MoDL, the best NRMSE and PSNR scores are achieved by the MHSA ViT architecture. For NMI, the MoDL score is the highest. The high Shannon entropy of all models, especially VarNet, imply the addition of redundant or distorting information. The lower entropy levels of the ViT reconstructions are preferable. Similarly, ViT achieves the best scores for EFC. Relative contrast is higher than the reference for all methods, suggesting a shift in intensity.

ANOVA shows statistically significant differences between the assessed methods for all metrics except relative contrast. Table 4 shows the Bonferroni-corrected paired *t*-test results for significance. The advantages of the different architectures are all assessed as significant, while the score gaps between the MHSA and WBSA block pipelines are significant for NRMSE, PSNR, and NMI. Augmentation in MHSA ViT is shown to be a significant improvement for all metrics except Shannon entropy.

**Figure 3:**
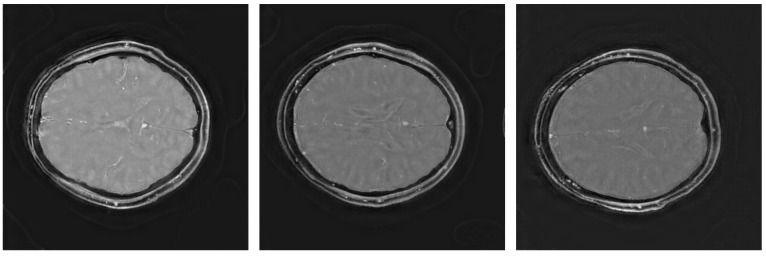
Triply augmented MHSA ViT reconstructions.

**Figure 4:**
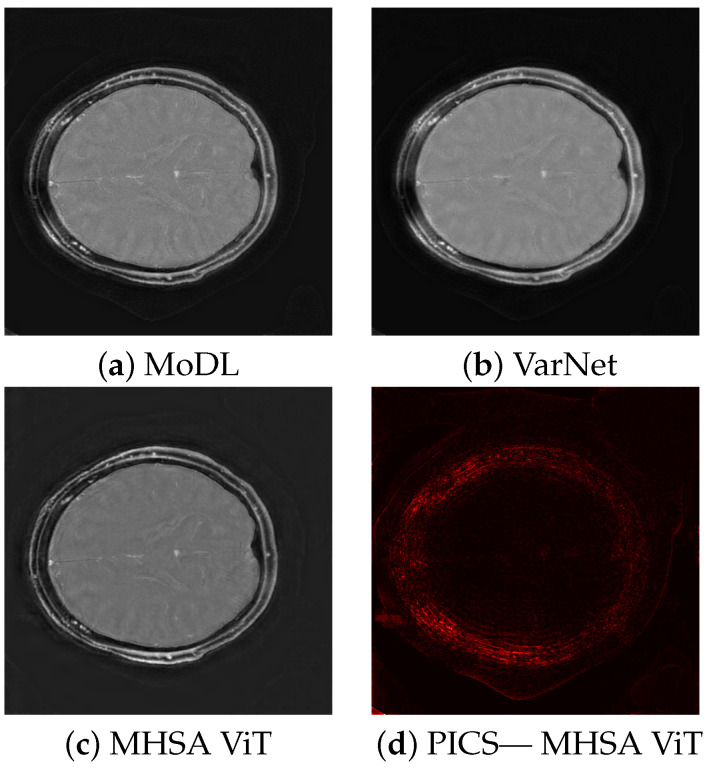
Comparison of reconstruction methods, showing results for MoDL, VarNet, MHSA ViT, and difference between PICS reference and MHSA ViT.

The quality scores demonstrate the strengths of the ViT network across multiple metrics. The triply augmented ViT achieves a respectable SSIM score while outperforming both VarNet and MoDL in terms of NRMSE and PSNR. Notably, the ViT results have better NRMSE and PSNR scores even with little or no augmentation to the training data. These result suggest that the ViT excels at preserving pixel intensity fidelity and reducing reconstruction noise. However, the lower NMI score indicates room for improvement in capturing fine inter-pixel relationships, particularly in regions of high structural complexity. Augmentation improves performance, with higher PSNR values demonstrating reduced noise and enhanced clarity. The lower Shannon entropy score for ViT suggests that a lesser amount of redundant information has been added during reconstruction. Likewise, the EFC scores indicate superior clarity for the ViT pipeline. Figure 3 illustrates sample triply augmented MHSA ViT results from the network. Figure 4 shows the same image reconstructed by VarNet, MoDL, and ViT pipeline; the final subfigure shows the amplified difference between the PICS and ViT reconstructions.

### 3.2. Network Runtime Performance

Table 5 highlights runtime and resources used for the models. The ViT pipelines demonstrate a marked advantage in processing speed, requiring approximately 60 s per 10 images, compared to VarNet and MoDL, which take approximately 7 min each. This efficiency arises from the network’s strategy of refining an already approximate reconstruction rather than computing directly from raw k-space data. We extrapolate from acquired data, assuming approximate linearity due to slice by slice processing, to provide estimates for processing a full 3D dataset comprised of 128 and 512 slices.

The GPU memory footprint of the ViT pipeline lies between VarNet and MoDL. While its resource requirements (4895 MB) exceed those of VarNet, it remains significantly lower than MoDL’s usage. Due to programming optimizations, the maximum total memory requirements remain the same regardless of slice count.

### 3.3. Noise Independence

**Figure 5:**
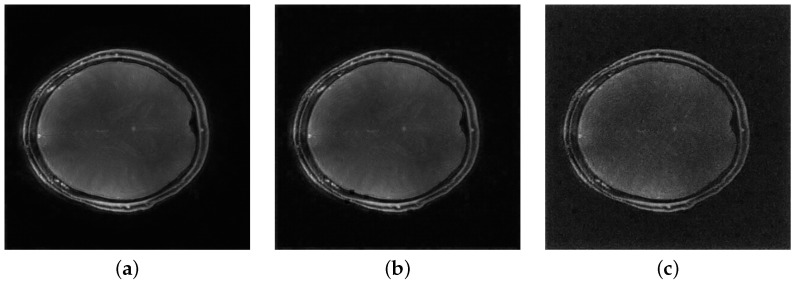
MHSA ViT reconstructions for noisy inputs. Values indicate Gaussian variance (**a**) Minimal noise ($5\times 10^{-12}$), (**b**) Mild noise ($1\times 10^{-11}$), (**c**) Moderate noise ($5\times 10^{-11}$).

Figure 5 shows the performance of the proposed ViT networks in the presence of k-space noise added with the BART toolbox. Gaussian variance levels were selected to provide minimal, mild, and moderate levels of noise with reference to the magnitude of the k-space data. The image quality metrics, listed in Table 6, show that while a large decay is observed for high noise conditions, the models tolerate a reasonable level of noise without excessive loss. Mild patch artifacts that occur at a variance of $1\times 10^{-11}$ indicate that the model is only robust up to a moderate level of corruption.

## 4. Discussion

The results of this study illustrate the potential of using a vision transformer pipeline for the reconstruction of rosette trajectory MRI data. The NRMSE and PSNR scores achieved against methods such as VarNet and MoDL suggest that ViT is effective in preserving image fidelity (NRMSE, PSNR) and reducing noise (PSNR). While interpreting entropy metrics is non-trivial, the Shannon entropy and EFC scores of ViT are preferable with respect to the principle of maximizing reconstruction fidelity. The competitive scores achieved with minimal or no augmentation highlight the ViT’s ability to generalize well from approximations and small datasets, which is crucial in medical imaging where large, well-annotated datasets are not always available. The SSIM and NMI scores indicate that while the method excels in certain metrics, there may be room for improvement in capturing fine structural details or maintaining overall structural integrity. The slight edge of the MSHA block over WBSA in this respect suggest that a global attention scope is preferable at this level of feature detection.

In addition to VarNet and MoDL, alternative AI-based methods such as RAKI and automap have shown success in MR image reconstruction, though they often require complex preprocessing steps to learn the inverse Fourier transformation [44]. Generative adversarial networks have likewise achieved impressive results with high quality and artifact reduction but are known to be unstable during training [45]. Vision transformers offer a balanced intermediate, with a predictable if moderately intensive training process and straightforward preprocessing, especially if combined with IFFT. This renders the model flexible in terms of development and deployment.

The noise independence experiments suggest that the model has a baseline resilience against noise, but the artifacts that arise at moderate Gaussian variance suggest that fine tuning or augmentation with noisy data could be advisable to improve performance under such conditions. The increased runtime efficiency of the framework suggests its suitability for real-time clinical applications, such as dynamic or 4D MRI, where processing speed is critical. However, structural fidelity remains a concern, especially for clinical contexts with subtle pathological features. The strong performance of the method at a resolution of 512 by 512 pixels suggests that features can be increased at lower resolutions to improve performance. Finally, although augmentation is possible, acquiring varied and high-quality primary datasets is often challenging for more complex clinical applications such as multimodal imaging. Performance under fine-tuning conditions is therefore an important avenue of investigation for future applications.

Investigating advanced augmentation techniques can improve the model’s generalization capabilities. The augmentation methods used, although simple (flipping, rotating, and color jitter), appear to have bolstered the model’s ability to generalize across different types of k-space data, as seen from the improved PSNR and NRMSE scores. Future work could investigate the impact of more complex augmentations, such as elastic transformations or non-uniform intensity scaling, which might further increase the effectiveness of the model across clinical scenarios.

While the results demonstrate strong performance on axial brain images, the generalizability of these findings to other MRI protocols (such as differing field strengths, acquisition times, or anatomical features) remains to be explored. Additionally, evaluating the ViT’s performance across different k-space trajectories, such as radial or spiral acquisitions, would further validate its effectiveness across different MRI applications. Incorporating more sophisticated initial approximations or iterative refinement steps within the ViT framework may also yield improvements. Finally, extending the evaluation of these methods to different types of MRI data, including dynamic and functional MRI, and assessing their performance in real-time applications will be crucial for translating these advancements into practical clinical tools. This approach could be particularly advantageous in exploring 3D or 4D MRI applications, as the transformer self-attention capability can naturally extend to higher dimensions.

## Figures and Tables

**Table 1:** Comparison of MRI reconstruction techniques for rosette trajectory imaging.

Technique	Advantages	Disadvantages
**IFFT [3]**	– Robust initial approximation from k-space to image domain. – Computationally efficient for uniformly sampled Cartesian data.	– Struggles with non-Cartesian trajectories. – Susceptible to artifacts due to irregular and undersampled data.
**CS [4,6]**	– Balances fidelity to acquired data with image sparsity. – Effective for undersampled data, reducing scan times. – Works well with Cartesian and non-Cartesian data.	– Requires complex optimization, leading to higher computational costs. – Sensitive to parameter tuning and model assumptions.
**VarNet [14,19], MoDL [20]**	– Learns complex mappings from data, improving reconstruction at high acceleration rates. – Handles both Cartesian and non-Cartesian data without regridding.	– Requires large datasets and computational resources for training. – Long inference times
**ViT [21,24]**	– Models long-range dependencies, capturing complex spatial patterns. – No need for extensive pre-processing of non-Cartesian data.	– Requires augmented training data, increasing training time and resource consumption.

**Table 2:** Formulae for image quality metrics, where **X** is the reconstructed region of interest and **Y** is the corresponding reference.

Metric	Formula
**SSIM**	$$\mathrm{SSIM}\left(X,Y\right)=\frac{\left(2\mu_{X}\mu_{Y}+c_{1}\right)\left(2\sigma_{XY}+c_{2}\right)}{\left(\mu_{X}^{2}+\mu_{Y}^{2}+c_{1}\right)\left(\sigma_{X}^{2}+\sigma_{Y}^{2}+c_{2}\right)}$$ where $\mu_{X}$ and $\mu_{Y}$ are the means of X and Y, $\sigma_{X}^{2}$ and $\sigma_{Y}^{2}$ are the variances, $\sigma_{XY}$ is the covariance, and $c_{1}$ and $c_{2}$ are constants to stabilize the division.
**NRMSE**	$$\mathrm{NRMSE}=\frac{\sqrt{\frac{1}{N}\sum_{i=1}^{N}{\left(x_{i}-y_{i}\right)}^{2}}}{\max(X)-\min(Y)}$$ where $x_{i}$ and $y_{i}$ are the pixel values of the reference and processed images, respectively, and *N* is the total number of pixels.
**NMI**	$\mathrm{NMI}=\frac{2I(X,Y)}{H(X)+H(Y)}$ where I(X,Y) is the mutual information between regions X and Y, and H(X) and H(Y) are the entropies of images X and Y, respectively.
**Relative Contrast**	$\mathrm{Relative}\;\mathrm{Contrast}=\frac{\max(X)-\min(X)}{\max(X)+\min(X)}$ where max(X) and min(X) are the maximum and minimum pixel intensities of the region.
**PSNR**	$\mathrm{PSNR}\left(X,Y\right)=10\log_{10}\left(\frac{L^{2}}{\mathrm{MSE}(X,Y)}\right)$ where *L* is the maximum pixel value and MSE(X,Y) is the mean squared error between X and Y.
**Shannon Entropy**	$$H\left(X\right)=-\sum_{i}p_{i}\log_{2}p_{i}$$ where $p_{i}$ represents the probability of intensity level *i* occurring in the image X.
**EFC**	$$E=-\sum_{j=1}^{N}\frac{x_{j}}{x_{\max}},x_{\max}=\sqrt{\sum_{j=1}^{N}x_{j}^{2}}$$ where $x_{i}$ represents pixel intensities in the image.

**Table 3:** Image quality scores for reconstruction methods. Arrows indicate whether higher or lower values are preferred.

Method	SSIM ↑	NRMSE ↓	PSNR ↑	NMI ↑	R. Contrast	Shannon	EFC ↓
Reference	-	-	-	-	0.332	3.840	2.960
VarNet	0.944	0.322	22.740	0.598	0.430	5.003	4.023
MoDL	**0.987**	0.060	37.248	**0.616**	0.472	4.861	3.429
Vision T.							
Non-aug. MHSA	0.974	0.048	40.134	0.501	0.441	4.697	3.244
Aug. MHSA (X1)	0.975	0.040	42.124	0.510	0.445	4.672	3.280
Aug. MHSA (X3)	0.980	**0.033**	**43.799**	0.536	0.445	**4.631**	**3.245**
Aug. WBSA (X3)	0.980	0.037	42.685	0.544	0.439	4.663	3.285

**Table 4:** Statistical significance (*p*-values) of pairwise comparisons between reconstruction methods. Significant differences are based on the Bonferroni-corrected threshold of α=0.0083.

Metric	MHSA(X3) vs. MoDL	MHSA(X3) vs. VarNet	WBSA(X3) vs. MoDL	WBSA(X3) vs. VarNet	MHSA(X3) vs. WBSA(X3)	MHSA X3 vs. X1
SSIM	*p* < 0.0083	*p* < 0.0083	*p* < 0.0083	*p* < 0.0083	***p*** ** > 0.0083**	*p* < 0.0083
NRMSE	*p* < 0.0083	*p* < 0.0083	*p* < 0.0083	*p* < 0.0083	*p* < 0.0083	*p* < 0.0083
PSNR	*p* < 0.0083	*p* < 0.0083	*p* < 0.0083	*p* < 0.0083	*p* < 0.0083	*p* < 0.0083
NMI	*p* < 0.0083	*p* < 0.0083	*p* < 0.0083	*p* < 0.0083	*p* < 0.0083	*p* < 0.0083
Shannon	*p* < 0.0083	*p* < 0.0083	*p* < 0.0083	*p* < 0.0083	***p*** ** > 0.0083**	***p*** ** > 0.0083**
EFC	*p* < 0.0083	*p* < 0.0083	*p* < 0.0083	*p* < 0.0083	***p*** ** > 0.0083**	*p* < 0.0083

**Table 5:** Reconstruction performance metrics.

Network	Total CPU Time (Hours:Minutes:Seconds)	Max GPU Memory Used (MB)
VarNet (10 slices)	00:06:58	2785
VarNet (20 slices)	00:13:51	
VarNet (estimate for 128 slices)	01:28:00	
VarNet (estimate for 512 slices)	05:54:00	
MoDL (10 slices)	00:07:05	6369
MoDL (20 slices)	00:14:08	
MoDL (estimate for 128 slices)	01:30:00	
MoDL (estimate for 512 slices)	06:00:00	
MHSA ViT (10 slices)	00:00:45	4895
MHSA ViT (20 slices)	00:01:25	
MHSA ViT (estimate for 128 slices)	00:09:00	
MHSA ViT (estimate for 512 slices)	00:36:00	
WBSA ViT (10 slices)	00:01:09	4895
WBSA ViT (20 slices)	00:01:26	
WBSA ViT (estimate for 128 slices)	00:10:00	
WBSA ViT (estimate for 512 slices)	00:37:00	

**Table 6:** Image quality scores for different noise levels and architectures. Arrows indicate whether higher or lower values are preferred.

ViT	Gaussian Variance	SSIM ↑	NRMSE ↓	PSNR ↑	NMI ↑
MHSA	$5\times 10^{-11}$	0.732	0.151	29.377	0.199
MHSA	$1\times 10^{-11}$	0.917	0.090	34.078	0.308
MHSA	$5\times 10^{-12}$	0.944	0.063	37.389	0.360
MHSA	$1\times 10^{-12}$	0.970	0.041	41.582	0.460
MHSA	No noise	0.980	0.033	43.799	0.536
WBSA	$5\times 10^{-11}$	0.745	0.118	31.702	0.200
WBSA	$1\times 10^{-11}$	0.822	0.203	27.471	0.259
WBSA	$5\times 10^{-12}$	0.955	0.053	39.139	0.385
WBSA	$1\times 10^{-12}$	0.974	0.041	41.509	0.484
WBSA	No noise	0.980	0.037	42.685	0.544

## Data Availability

The MRI data used in this study are available at https://drive.google.com/drive/folders/1lTaz1xMRGX6jEKLzU6-r6ifaOo7UVTwI?usp=sharing (accessed on 10 March 2025).
